# A Solution to Modeling Multilevel Confirmatory Factor Analysis with Data Obtained from Complex Survey Sampling to Avoid Conflated Parameter Estimates

**DOI:** 10.3389/fpsyg.2017.01464

**Published:** 2017-09-22

**Authors:** Jiun-Yu Wu, John J. H. Lin, Mei-Wen Nian, Yi-Cheng Hsiao

**Affiliations:** ^1^Institute of Education, National Chiao Tung University Hsinchu, Taiwan; ^2^Office of Institutional Research, National Central University Taoyuan, Taiwan

**Keywords:** multilevel confirmatory factor analysis, design-based approach, model-based approach, maximum model, level-varying factor loadings, complex survey sampling, measurement

## Abstract

The issue of equality in the between-and within-level structures in Multilevel Confirmatory Factor Analysis (MCFA) models has been influential for obtaining unbiased parameter estimates and statistical inferences. A commonly seen condition is the inequality of factor loadings under equal level-varying structures. With mathematical investigation and Monte Carlo simulation, this study compared the robustness of five statistical models including two model-based (a true and a mis-specified models), one design-based, and two maximum models (two models where the full rank of variance-covariance matrix is estimated in between level and within level, respectively) in analyzing complex survey measurement data with level-varying factor loadings. The empirical data of 120 3rd graders' (from 40 classrooms) perceived Harter competence scale were modeled using MCFA and the parameter estimates were used as true parameters to perform the Monte Carlo simulation study. Results showed maximum models was robust to unequal factor loadings while the design-based and the miss-specified model-based approaches produced conflated results and spurious statistical inferences. We recommend the use of maximum models if researchers have limited information about the pattern of factor loadings and measurement structures. Measurement models are key components of Structural Equation Modeling (SEM); therefore, the findings can be generalized to multilevel SEM and CFA models. Mplus codes are provided for maximum models and other analytical models.

## Introduction

Multilevel Confirmatory Factor Analysis (MCFA) extends the power of Confirmatory Factor Analysis (CFA) to accommodate the complex survey data with the estimation of the level-specific variance components and the respective measurement models. Complex survey data are obtained through cluster sampling or multistage sampling, where a few individuals within a class/household or the entire class/family are selected. This type of sampling scheme is likely to result in non-independent observations with within-cluster dependency (Skrondal and Rabe-Hesketh, 2007). If the dependent data are analyzed through the traditional approaches which assume independent observations, “incorrect parameter estimates, standard errors, and inappropriate fit statistics may be obtained” (du Toit and du Toit, 2008, p. 456).

Researchers has devoted their attention in discussing the influences of applying different multilevel modeling constructions on complex survey data (e.g., model-fit indices: Hsu et al., 2015; reliability measures: Geldhof et al., 2013; parameter estimates and statistical inferences: Wu and Kwok, 2012; longitudinal design: Wu et al., 2014). Among the research designs in these studies, the issue of inequality in the between- (i.e., the higher level or cluster level) and within-level (i.e., the lower level or individual level) structure in complex survey data has been proven to be influential for obtaining unbiased parameter estimates along with their consistent statistical inferences. Compared to inequality of level structures in multilevel models, a less addressed condition is that the true model did have the same factor structure at both levels while the magnitudes and statistical significance of the factor loadings varied across levels and varied within the levels, which occurred frequently in empirical research (e.g., Dyer et al., 2005; Klangphahol et al., 2010).

For example, Dyer et al. (2005) applied MCFA to study organizational leadership at the individual and societal level and obtained a common factor consisting of five items of being “formal,” “habitual,” “cautious,” “procedural,” and “ritualistic.” The five items loaded much stronger onto the single factor at the between level (i.e., societal level) than at the within level (individual level), which supported the belief that this leadership scale operates mainly at the societal level. Based on this finding, Dyer et al. (2005) suggested that a three-item factor (discarding two trivial items with small factor loadings) instead of a five-item factor should be used if the interest of leadership study is at the individual level. Dyer et al.'s suggestion capitalized on the importance of specifying an optimal measurement model with complex survey data in terms of both model structure and sizes of factor loadings to obtain correct statistical and practical interpretations in scale development.

From the factor analysis point of view, items with variance explained smaller than 20% or standardized factor loadings less than 0.45 would be considered as low communality (EFA: MacCallum et al., 1999; CFA: Meade and Bauer, 2007). From a measurement point of view, items with standardized factor loadings larger than 0.6 would exhibit better psychometric properties (Bagozzi and Yi, 1988; Kline, 2010). Failing to detect items with small factor loadings may lead to a misunderstanding that all items are equally important, causing researchers to investigate problems that are of little importance or little relevance to the intended measure.

Therefore, in this study, we performed a substantive-methodological synergy (Marsh and Hau, 2007) by applying different modeling strategies on simulated synthetic datasets with population parameters specified based on an empirical dataset to examine the robustness of model-based, design-based, and maximum models regarding their effectiveness and efficiency in producing unbiased parameter estimates and statistical inference for the measurement data obtained from complex survey sampling. Below we elaborated on the issues with modeling strategies and unequal factor loadings, followed by introduction to three modeling strategies on complex survey data.

### Issues with modeling strategies and unequal factor loadings

Traditionally, several multilevel modeling strategies can be applied to address data dependency in complex survey data (Heck and Thomas, 2008; Rabe-Hesketh and Skrondal, 2008; Hox, 2010; Snijders and Bosker, 2011). Specifying different structures for separate levels, namely a model-based approach, on complex survey data allows free estimation of level-specific parameters and enables the detection of possible inequality in parameter estimates. However, in reality, information or truth about the higher-level structure is rarely known without the support of theoretical evidence. If researchers jump into multilevel analysis without theoretical or empirical evidence, the correctness of the multilevel structure is at risk.

Alternatively, researchers can apply the design-based approach by specifying only an overall model for the complex survey data to infer their findings to the lower level sampling units, and using the robust standard error estimator (Huber, 1967; White, 1980) to correct for the bias in standard error of the fixed effects (Muthén and Satorra, 1995). The design-based approach has been proved to yield satisfying analytic results only when the complex survey data meet the assumption of equal structures in both between- and within-levels (Wu and Kwok, 2012). In addition to design-based and model-based approaches, a possible alternative for analyzing multilevel data is through the use of maximum models (Hox, 2002, 2010; Wu and Kwok, 2012), where a saturated between-level model is estimated and can be used to focus on a specific level of analysis.

To examine the robustness of reliability measures on complex survey data, Geldhof et al. (2013) used MCFA and single-level CFA (i.e., without taking data dependency into consideration) on the simulated multilevel datasets, where the between and within levels had exactly the same factor loadings but with different high and low reliability across levels using the average item ICC as a dependency measure. Their study findings suggested that single-level CFAs cannot yield the actual scale reliability unless the true reliabilities are identical at each level. Moreover, in the simulation study, they postulated that the true MCFA model had the same factor loadings within and across levels, i.e., the between and within level model were identical in terms of magnitude of factor loadings and factor structures. Few studies have investigated the issue of inequality of factor loadings under equal factor structure within and across levels. Besides, systematic investigation on the performance of model-fit statistics, indices and information criteria, and the resulted parameter estimates with statistical inferences were not discussed in Geldhof et al. (2013).

Extending the simulation settings of Geldhof et al. (2013), we examined performance of different model specifications regarding the issues of inequality of factor loadings and different factor structures within and across levels. Distinct Cluster Numbers (CN), Cluster Sizes (CS) and ICCs were used in true model for the simulation settings of this study. The criterion variables include overall exact model fit chi-square test and various model fit indices, both fixed-effect and random-effect parameter estimates, their 95% coverage rate and empirical power, as well as the variance explained measure (*R*^2^) and scale reliability (ρ).

Specifically, this study aims to examine the robustness of the three modeling strategies using five analytic models (i.e., MCFA, miss-specified MCFA, one-level design-based CFA, Max CFA with saturated Between level, Max CFA with saturated Within level) in testing the multilevel measurement data with unequal magnitudes of factor loadings. Of the factor loadings, some may be trivial or of little relevance in a practical sense at the individual level under equal level structures. In the following section, we provide a review of three multilevel modeling strategies.

### Three modeling strategies on complex survey data

#### Model- and design-based strategies

The rationale for using multilevel models in analyzing complex survey data is to reflect the natural multistage sampling scheme (Muthén, 1994; Heck and Thomas, 2008). Researchers can do so by constructing the analytic model either to simultaneously calculate the lower- and higher-level parameter estimates which may have different values at each level or to adjust the standard errors of fixed effects. The model-based approach (e.g., MCFA technique) conforms to the actual multi-stage sampling scheme by specifying a level-specific model for each level of the data. In other words, for a two-level clustered sampling data, it specifies a between-level model that conforms to the level 2 structure (i.e., higher level) and a within-level model that conforms to the level 1 structure (i.e., lower level). Instead of constructing separate level models for multilevel data, the design-based approach analyzes the data with only one overall model and considers the sampling scheme by adjusting for the standard errors of the parameter estimates based on the sampling design. The adjustment is implemented using the robust standard error estimator (Huber, 1967; White, 1980) or sandwich-type variance estimator, a general name for alternative variance estimators. The sandwich-type variance estimator functions as an overall adjustment of the deviated standard error of parameter estimates due to extra data dependency along with the original statistical approach. This kind of relative variance estimators has been proposed to address data non-independence (i.e., data heteroskedasticity) more directly in CFAs (Muthén and Satorra, 1995). The adjustment is a *post-hoc* process and is said to only affect the standard errors, not the parameter estimates (Hardin and Hilbe, 2007).

In a simulation study, Muthén and Satorra (1995) showed that under the same model structure for all data levels, these two approaches performed equally well for complex survey data. Compared to the model-based approach, the design-based approach is used more frequently by researchers in the applied areas (Rebollo et al., 2006; Róbert, 2010; Roberts et al., 2010; Rosenthal and Villegas, 2010; Wu et al., 2010; Brook et al., 2011; Martin et al., 2011; Wu, 2015, 2017) because it only requires a single model specification and often researchers were interested in examining the lower level (i.e., the within-level) model with the most sampling units.

Despite the simplicity of the model's specifications, the design-based approach for complex survey data is built upon the assumption of the same level-varying structures (Muthén and Satorra, 1995; Wu and Kwok, 2012). However, this assumption is often violated in empirical research when researchers examine the level-specific structures of their multilevel dataset (e.g., Wilhelm and Schoebi, 2007). Inequality in the between- and within-level structures leads to conflated estimations of the fixed and random effects if the design-based approach is used (Wu and Kwok, 2012). What's more, in the current study, we posit that if the same magnitude and significance of factor loadings do not hold at different levels under same level structures, inequality of the between- and within-level factor loadings may also cause potential problem with the design-based approach. In the case of Dyer et al. (2005), if the authors had used the design-based approach for their procedural leadership analysis, they would obtain the design-based estimates which would have been contaminated with information from both the between- and within-level models. Thus, they would have no idea of the larger factor loadings at the societal level and may not be able to detect the two trivial items at the individual level. From a practical perspective, researchers would falsely conclude the scale is a valid measurement for the research question related to the individual participant. In addition, the estimation of the overall model parameters and the scale reliability measures may be questionable to infer the individual-level characteristics. However, the issue of inequality of factor loadings between and within the levels has rarely been systematically examined in previous studies.

#### Maximum model

Another feasible modeling strategy for complex survey data is called the “maximum model,” (Hox, 2002, 2010; Wu and Kwok, 2012) where a saturated model in specific level (usually the higher-level) is built by estimating the full rank of between-level variance-covariance matrix with the consumption of all available degrees of freedom. This maximum model technique was firstly suggested by researchers (e.g., Hox, 2002; Stapleton, 2006; Yuan and Bentler, 2007) as the baseline model for constructing multilevel analysis with theoretical evidence. Ryu and West (2009), on the other hand, examined the performance of level-specific fit indices using maximum modeling technique. More recently, Wu and Kwok (2012) found that the maximum model and correctly specified model-based approaches performed equally well for analyzing complex survey data regardless of equality in level structures whereas the design-based approach only produced satisfying fixed-effect estimates and standard error under equal within-/between-level structure scenarios. Compared to inequality of level structures, what is more commonly found in empirical measurement research is unequal magnitudes of factor loadings in different levels with the same number of factors. However, no study to date has systematically examined the consequences of miss-specifying multilevel models for a two-level CFA measurement data regarding the violation of equality of factor loadings. This study will focus on inequality of factor loadings within and across levels of MCFA to explore potential analytical problems.

In the SEM framework, analysts commonly use differential chi-square tests to conduct model comparison analysis with numerous completing models. However, this kind of test is only good for comparisons between nested models. Besides, the chi-square test statistic is easily influenced by large sample sizes (Yuan et al., 2007; Kline, 2010). Alternatively, information criteria statistics can be used for model comparison between nested and non-nested models (Sclove, 1987). By taking the model uncertainty into consideration, the information criteria overcome the above-mentioned difficulties (Bollen et al., 2014). In this study, besides commonly-used model-fit test statistics and indices, we discussed the performance of Akaike Information Criterion (AIC, Akaike, 1974), Bayesian/Schwartz Information Criterion (BIC, Schwarz, 1978), and the sample-size adjusted BIC (adj. BIC, Sclove, 1987) in assessing the different model specifications. Models with smaller AIC, BIC, or adjusted BIC would be considered a better fit to the designated dataset. Detailed discussion among these information criteria under the SEM framework can be found in Nylund et al. (2007) for Latent Class Analysis and Growth Mixture Modeling, and Bollen et al. (2014) for single-level SEM modeling. This study would add to the literature regarding the guideline of interpreting information criteria to construct measurement models for complex survey data under the SEM framework.

## Methods

### Mathematical investigation of three SEM techniques on complex survey measurement data

We provided the model specifications of the model-based, design-based, and maximum modeling approaches (with both saturated between-level model, and saturated within-level structure model) and their mathematical derivations to investigate the robustness of these modeling approaches in dealing with the inequality of factor loadings at between- and within-level models under equal factorial structures.

Using multilevel data drawn from a two-level multistage sampling strategy as an example, let us suppose that the *G* groups are randomly drawn from the target population at the first stage of sampling and that *n*_*g*_ participants are sampled within each group *g* at the second stage. We have a total of $N=\sum_{g=1}^{G}n_{g}$ participants. For each participant, *P* item responses (*y*_*pig*_, *p* = 1,2,…,*P*) are gathered. We now have random vector of response variables **y**_*ig*_ = [*y*_1*ig*_*, y*_2*ig*_*,…, y*_*pig*_]_1 × *P*_ for participant *i* (lower-level unit, *i* = 1,2,…,*n*_*g*_) within group *g* (higher-level unit, *g* = 1,2,…,*G*).

For the *g*th group, the random matrix of observations may be arranged as follows:

(1) $$y_{g}=\left[\begin{array}{c} y_{1g}\\ y_{2g}\\ \vdots \\ y_{n_{g}g} \end{array}\right]={\left[\begin{array}{c} \left[\begin{array}{cccc} y_{11g} & y_{21g} & \cdots & y_{P1g} \end{array}\right]\\ \left[\begin{array}{cccc} y_{12g} & y_{22g} & \cdots & y_{P2g} \end{array}\right]\\ \begin{array}{cccc} \vdots & \vdots & \cdots & \vdots \end{array}\\ \left[\begin{array}{cccc} y_{1n_{g}g} & y_{2n_{g}g} & \cdots & y_{Pn_{g}g} \end{array}\right] \end{array}\right]}_{n_{g}\times P}$$

Analogous to the variance decomposition used in ANOVA analysis, the observation **y**_*ig*_ can be decomposed into its between-group component and within-group component, that is,

(2) $$y_{ig}=y_{B\ldots g}+y_{W.ig},\forall i=1,2,\ldots ,n_{g},g=1,2,\ldots ,G$$

where **y**_*B*…*g*_ is the between-group component with *MVN*(**μ**, **Σ**_**B**_) (i.e., multivariate normal distribution with grand mean **μ** and variance-covariance matrix **Σ**_**B**_) and **y**_*W*.*ig*_ is the within-group component with *MVN*(**μ**, **Σ**_**W**_). Typically, **μ**_*g*_ is set as 0. The between-group components in different groups is set to be uncorrelated; that is, $Cov\left({\text{y}}_{B\ldots g},{\text{y}}_{B\ldots g^{\prime }}\right)=0$, ∀*g* ≠ *g*′. Similarly, the correlation between different participants in different groups is also set to be zero (i.e., $Cov\left({\text{y}}_{W.ig},{\text{y}}_{W.i^{\prime }g^{\prime }}\right)=0$, ∀*i* ≠ *i*′ & ∀*g* ≠ *g*′). Furthermore, the cross-level correlation between **y**_*B*…*g*_ and **y**_*W*.*ig*_ is defined as uncorrelated.

Hence, the variance-covariance matrix of **y**_*ig*_may be decomposed into the combination of between-group and within-group variations, *Cov*(**y**_*ig*_) = **Σ**_*B*_ + **Σ**_*W*_. Going a step further, to consider the MCFA model (i.e., the model-based approach), Equation (2) may be written as

(3) $$\begin{array}{l} y_{ig}=y_{B\ldots g}+y_{W.ig}\\ \;=\mu +\Lambda_{B}\eta_{B\ldots g}+\varepsilon_{B\ldots g}+\Lambda_{W}\eta_{W.ig}+\varepsilon_{W.ig} \end{array}$$

The between-group component **y**_*B*…*g*_ is the combination of a product of factor loading matrix **Λ**_*B*_ and latent factor **η**_*B*…*g*_~*MVN*(**0**, **Ψ**_**B**_), and the unique vector **ε**_*B*…*g*_~*MVN*(**0**, **Θ**_**B**_). The within-group component **y**_*W*.*ig*_ is the combination of a product of factor loading matrix **Λ**_*W*_ and latent factor **η**_*W*.*ig*_~*MVN*(**0**, **Ψ**_**W**_), and the unique vector **ε**_*W*.*ig*_~*MVN*(**0**, **Θ**_**W**_). Random components were set to be orthogonal (i.e.,**η**_*B*…*g*_⊥**ε**_*B*…*g*_⊥**η**_*W*.*ig*_⊥**ε**_*W*.*ig*_).

Equation (3) specifies two sources of random variation for the observed variables, within-group (i.e., within-level) variation and between-group (i.e., between-level) variation to the nature of complex survey data, rather than just one overall random source. As a result, the variance-covariance matrix of **y**_*ig*_ may be further rewritten as

(4) $$\begin{array}{l} Cov\left(y_{ig}\right)=Cov(\mu_{B}+\Lambda_{B}\eta_{B\ldots g}+\varepsilon_{B\ldots g}+\mu_{W}+\Lambda_{W}\eta_{W.ig}\\ \;+\;\varepsilon_{W.ig})\\ \;=Cov\left(\Lambda_{B}\eta_{B\ldots g}+\varepsilon_{B\ldots g}\right)+Cov\left(\Lambda_{W}\eta_{W.ig}+\varepsilon_{W.ig}\right)\\ \;=\Lambda_{B}\Psi_{B}\Lambda_{B}\prime +\Theta_{B}+\Lambda_{W}\Psi_{W}\Lambda_{W}\prime +\Theta_{W}\;(\text{MCFA}) \end{array}$$

The variance covariance matrix of indicators is a function of random effects and fixed effects in both between- and within-level models. Using the multilevel CFA model, the total variance-covariance of observations may be expressed as a combination of three components in two levels: (a) factor loadings between indicators and latent factors (**Λ**_*B*_ and **Λ**_*W*_), (b) latent factor variances and covariance (explained portion of observed variance, **Ψ**_*B*_ and **Ψ**_*W*_), and (c) residual variance of indicators (unexplained portion of observed variance, **Θ**_*B*_ and **Θ**_*W*_). The single-factor intraclass correlation (ICC) of MCFA is then defined as **ICC** = **Ψ**_B_(**Ψ**_**B**_ + **Ψ**_**W**_)^−1^ (Muthén, 1991, 1994).

When the maximum modeling technique is applied to analyze these two-level data, Equation (4) becomes

$$\begin{array}{l} Cov\left(y_{ig}\right)=\Sigma_{B}^{Saturated}+\Lambda_{W}\Psi_{W}\Lambda_{W}\prime +\Theta_{W}\\ \;(\text{Max CFA with saturated between}\\ \text{ -level structure},\text{ 5}.\text{1}) \end{array}$$

or

$$\begin{array}{l} Cov\left(y_{ig}\right)=\Lambda_{B}\Psi_{B}\Lambda_{B}\prime +\Theta_{B}+\Sigma_{W}^{Saturated}\\ \;(\text{Max CFA with saturated within}\\ \text{ -level structure},\text{ 5}.\text{2}) \end{array}$$

The full-rank variance-covariance matrix $\Sigma_{B}^{Saturated}$ or $\Sigma_{W}^{Saturated}$ is unstructured, that is, all the possible between-level or within-level variation of indicators is estimated and separated from their total variance component. For the multilevel measurement model specification in this study, the unique within-level or between-level variation is then used to construct the respective within-level or between-level model with fixed and random effects without contamination from the other level. The residual part is the unique portion of total variation to the within-level or between-level variation of the indicators. If **Λ**_*B*_ = **Λ**_*W*_ (i.e., equality of factor loadings and structures holds for between-/ within-level models), the resulting factor loading estimates of design-based approach with one-level model are equal to the between-/within-level factor loadings in the true two-level model (i.e., $\Lambda_{B}\text{ =}\Lambda_{W}\text{ =}\Lambda_{y}$).

If we ignore the multilevel structure and construct a one-level model with design-based approach for the multilevel dataset **y**, the observed variance-covariance matrix of the indicators may be represented with the model-driven parameters as follows:

(6) $$\begin{array}{l} Cov\left(y\right)=\Lambda_{y}\Psi \Lambda_{y}\prime +\Theta_{\varepsilon }=\Lambda_{y}\left(\Psi_{B}+\Psi_{W}\right)\Lambda_{y}\prime \\ \;+\left(\Theta_{B}+\Theta_{W}\right)(\text{1-level CFA}) \end{array}$$

With the inclusion of ICC, Equation (4) can be further reformatted as:

(7) $$\begin{array}{l} Cov\left(y\right)=\Lambda_{B}^{\prime }\;ICC\;\Psi \Lambda_{B}^{\prime }+\Lambda_{W}\left(I-ICC\right)\Psi \Lambda_{W}^{\prime }\\ \;+\left(\Theta_{B}+\Theta_{W}\right) \end{array}$$

However, if the magnitudes of non-zero elements in between-/within-level factor loading matrix are not the same, the factor loading estimates of design-based approach is a function of true between- and within-level factor loadings and the ICC measures. Snijders and Bosker (2011) shows that, in univariate case, the regression coefficient of overall model with multilevel dataset will be λ_*y*_ = *ICCλ*_*B*_ + (1 − *ICC*)λ_*W*_. In the MCFA case, if there is a uni-factor structure in both levels, we hypothesize that the factor loading estimates of design-based approach could be simplified as (which is later being validated by the simulation result):

(8) $$\Lambda_{y}=ICC\Lambda_{B}+\left(1-ICC\right)\Lambda_{W}$$

That is, design-based approach could yield a conflated factor loading estimate (**Λ**_*y*_) of complex survey data. If the indicator has more variation in the within level, its factor loading estimate from design-based approach will be close to its within-level counterpart; if the indicator has more variation in the between level, its conflated factor loading estimate will be close to its between-level counterpart.

The composite reliability with congeneric measures based on CFA can then be calculated for the above models (Raykov, 2004; Brown, 2006), using:

(9) $$\rho \text{ = }{\left(\sum_{\text{p}=1}^{\text{P}}\lambda_{\text{p}}\right)}^{\text{2}}/\left[{\left(\sum_{\text{p}=1}^{\text{P}}\lambda_{\text{p}}\right)}^{\text{2}}\text{+}\sum_{\text{p}=1}^{\text{P}}\Theta_{\text{p}}\right],$$

where λ_*p*_ is the factor loading of item *p* onto a single common factor and Θ_*p*_ is the unique variance of item *p*. When constructing a one-level model, we can insert Equation (8) into (9) to obtain the reliability for the design-based model in Equation (10), which can be further expressed as the function of between- and within-level factor loadings and errors:

(10) $$\rho_{Design-Based\;Approach}=\frac{\left[{\left(\sum_{p=1}^{P}ICC\lambda_{Bp}\right)}^{2}+{\left(\sum_{p=1}^{P}\left(1-ICC\right)\lambda_{Wp}\right)}^{2}\right]}{\left\{\left[{\left(\sum_{p=1}^{P}ICC\lambda_{Bp}\right)}^{2}+{\left(\sum_{p=1}^{P}\left(1-ICC\right)\lambda_{Wp}\right)}^{2}\right]+\sum_{p=1}^{P}\left(\Theta_{Bp}+\Theta_{Wp}\right)\right\}},$$

Where λ_*BP*_ and λ_*Wp*_ are the standardized factor loadings of item *p* in the between- and within-level, and Θ_*BP*_ and Θ_*Wp*_ are residual variances of item *p* in the between- and within-level. The detailed discussion about reliability measures in complex survey data with MCFA and CFA can be referred to Geldhof et al. (2013).

In the following sections, the simulation study was provided to illustrate the robustness of the three SEM modeling strategies with five model specifications in analyzing a measurement dataset obtained from complex survey. The simulation results could inform the influences of different modeling techniques on overall exact model fit chi-square test and various model fit indices, information criteria, parameter and standard error estimates as well as the statistical inferences in the statistical analysis. Parameter Specification for the Simulation From a substantive-methodological synergy (Marsh and Hau, 2007) perspective, we specified the population parameters in our simulations based on the parameter estimates obtained from an empirical dataset to examine the performance of the proposed modeling approaches on multilevel measurement data.

### Empirical dataset: measurement and sampling

From a sample of 784 academically at-risk children participating in a longitudinal study, we selected a balanced dataset of 120 students nested within 40 classrooms with 3 students in each class. A total of 120 students (47 Females and 73 males; 39 African Americans, 38 Hispanics, 40 Caucasians and 3 Asians/Pacific Islanders) were drawn. No evidence of selective consent for participation in the larger longitudinal study was found. Details about recruitment of multilevel sampling procedure of the 784 participants were reported in Hughes and Kwok (2007). Their Grade 3 Harter competence measures were used in the current study. We generated the balanced-design synthetic datasets based on the parameter estimates from the MCFA of their Harter competence measures, considering different levels of cluster sizes, cluster numbers and intraclass correlations.

The Children Perceived Competence Scale (CPC, Harter, 1982) is composed of three domain-specific competences, including child-perceived competence in scholastic competence (CPCSC), social acceptance (CPCSA), and athletic competence (CPCAC), as well as a general global self-worth scale (CPCSW). The item-level responses consisted of ordered and categorical 4-point scale. Each of the subscale was measured using 7 items for a total of 28 items. Reliability of the item-level subscales ranged from 0.75 to 0.86. We used the composite scores of each subscale to form four continuous indicators for children's general competence at both classroom and individual levels so that the analysis result can be generalized to continuous responses.

### Simulation study: true model specification

**Figure 1:**
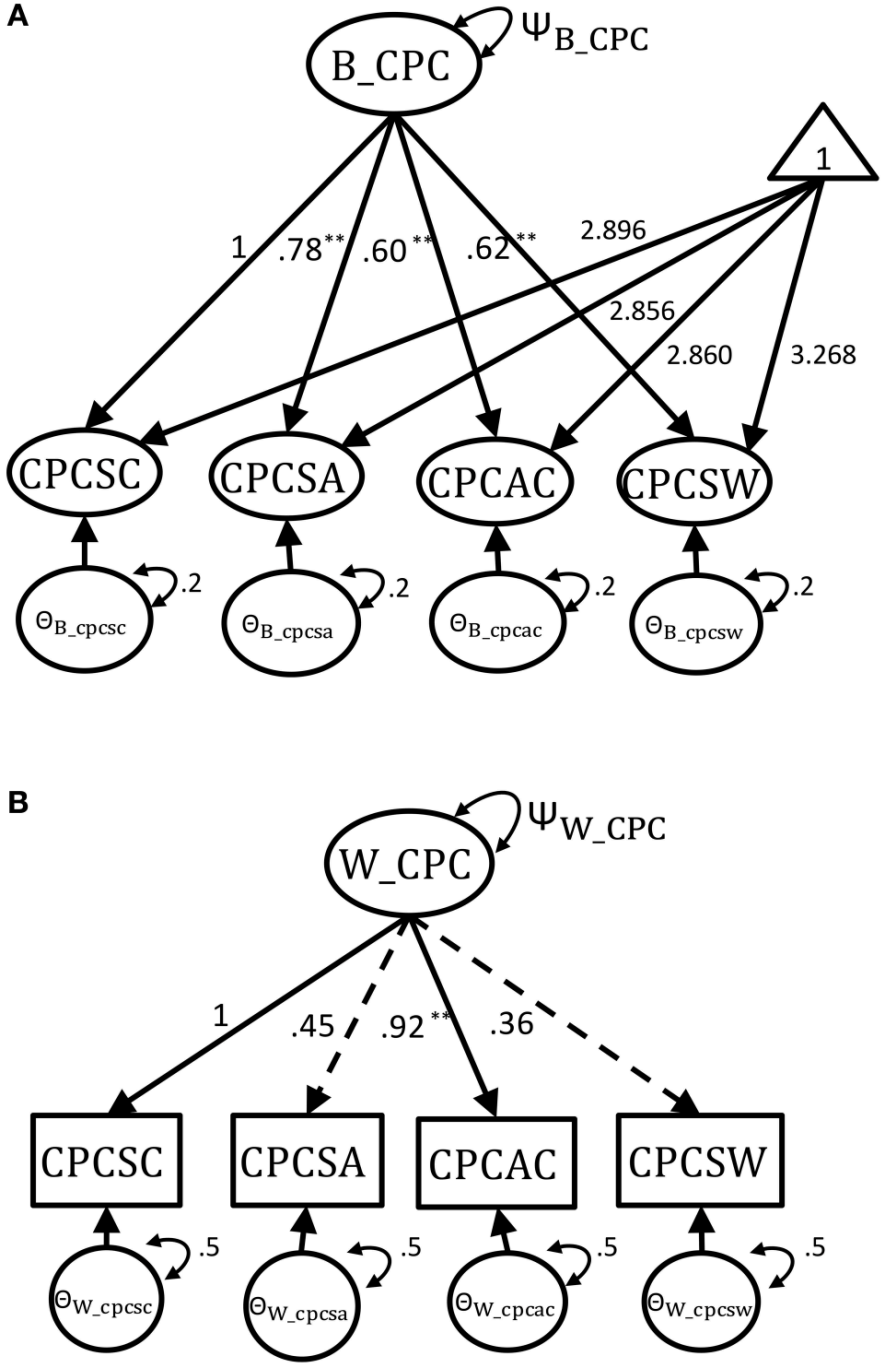
The multilevel CFA model with parameters from empirical Harter dataset. **(A)** The true between-level model. **(B)** The true within-level model. ^**^p < 0.05.

In order to demonstrate the adequacy and robustness of five different modeling approaches, we used Monte-Carlo simulation to generate the synthetic complex survey dataset with known true multilevel measurement model of CPC scale. A two-level uni-factor CFA model was firstly built for the empirical dataset of CPC scale with an overall factor of child-perceived competence including three domain-specific subscale indicators and one general self-worth indicator in both the between- and within-levels for the empirical dataset (as shown in Figure 1). With Full Information Maximum Likelihood (FIML) estimation, the resulting two-level CFA has an adequate model fit test statistic and index values (χ^2^ = 9.421 with *df* = 4 and *p* = 0.051, *CFI* = 0.990, *RMSEA* = 0.048, *SRMR-Within* = 0.023, *SRMR-Between* = 0.018). The parameter estimates of varying factor loadings were retained in the true models for simulation. The ICCs for the indicators in the empirical analysis ranged from 0.352 to 0.617. The factor variances in between- and within-level would then be altered to have different ICC settings in the simulation study.

Even though the between- and within-level model had equal structures, their factor loading magnitudes and patterns of significance were distinct for this empirical dataset (see the two dashed lines in Figure 1B). The unstandardized factor loading estimates from the two-level CFA analysis of empirical dataset were used as the population values for Monte Carlo simulation. The population values for the within-level factor loadings was 1 for scholastic competence (marker variable with standardized factor loading λ = 0.719), 0.45 for social acceptance (λ = 0.400), 0.92 for athletic competence (λ = 0.694), and 0.36 for global self-worth (λ = 0.331). In the within-level, only athletic competence was a statistically significant factor loading (i.e., *p* ≤ 0.05). On the other hand, all the between-level factor loadings were statistically significant. The between-level factor loadings were 1 (marker variable with standardized factor loading λ = 0.910) for scholastic competence, 0.78 for social acceptance (λ = 0.871), 0.60 for athletic competence (λ = 0.807), and 0.62 for global self-worth (λ = 0.816). The intercepts of the indicators were set as 2.896 for scholastic competence, 2.856 for social acceptance, 2.860 for athletic competence, and 3.268 for global self-worth. Finally, the population values of residual variance for the classroom- and individual-level indicators were set as 0.2 and 0.5. Total variance of factor was set at one (Ψ_CPC_ = Ψ_B_CPC_ + Ψ_W_CPC_ = 1), and the between- and within-level factor variance was set as Ψ_B_CPC_ and Ψ_W_CPC_. The levels of intraclass correlation (ICC) were then manipulated as Ψ_B_CPC_**(**Ψ_B_CPC_ + Ψ_W_CPC_**)**^−1^. For the simulation study, the true two-level model was constructed with these empirical parameter estimates under varying conditions of cluster size (CS = 3, 30, 200), cluster number (CN = 40, 100, 300) and Intraclass correlation (ICC = 0.1, 0.3, 0.5, 0.7, 0.9, Muthén, 1994) to generate 1,000 converged copies of balanced-design complex survey datasets. A total of 3(CS)^*^ 3(CN)^*^5(ICC)^*^1,000(reps) = 45,000 synthetic multilevel datasets were generated.

### Simulation study: analytical models specification

**Figure 2:**
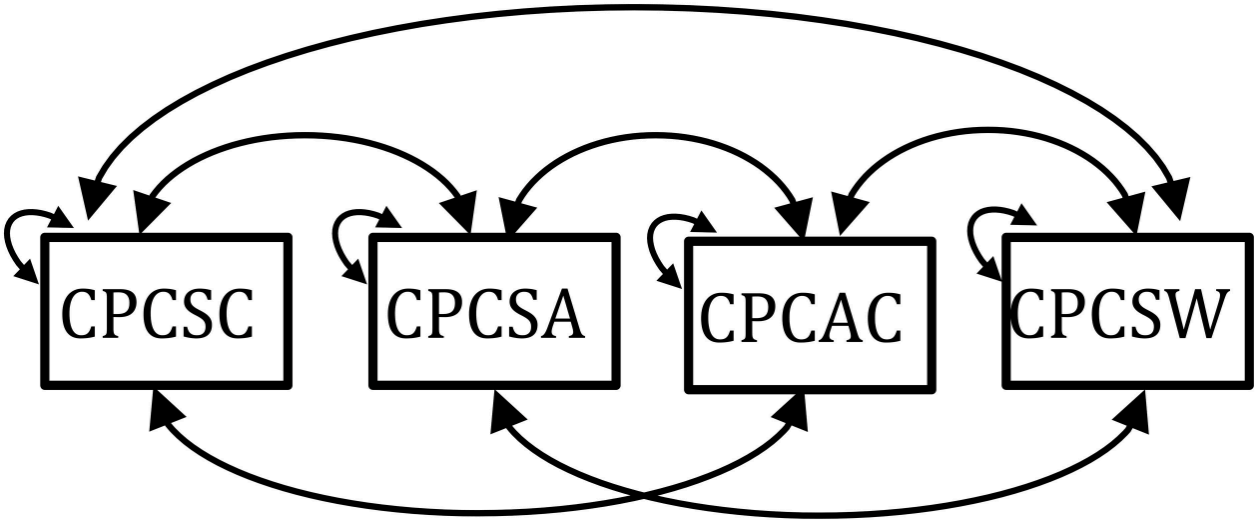
The saturated model.

Five SEM models for multilevel data with robust estimation were used to analyze the synthetic datasets. For ease of differentiation, we used the following naming scheme for the five model specifications:

1. 2MLR: the two-level model-based model and the true model (Figures 1A,B).
2. 1MLR: the one-level design-based model (Figure 1B).
3. 2MaxB^1^: the two-level maximum model with saturated model in between level (Figure 2) and true model in within level (Figure 1B).
4. 2MaxW: the two-level maximum model with true model in between level (Figure 1A) and saturated model in within level (Figure 2).
5. 2Miss: the miss-specified two-level model was constructed as Figures 1A,B by constraining the factor loading estimates of the between and within levels to be the same. This miss-specified model was used to test if the model-based approach is robust in detecting trivial items, and to examine if this model performs the same as design-based approach (i.e., 1MLR).

Two Mplus built-in routines were employed for the statistical modeling (Muthén and Muthén, 2012). First, the TYPE = TWOLEVEL routine, which allows level-specific specifications for complex survey data, was used for the 2MLR, 2MaxB, 2MaxW, and 2Miss). Second, TYPE = COMPLEX was used as design-based approach, where only a single level model is estimated (i.e., 1MLR) for complex survey data. By default, both routines use the full information maximum likelihood (FIML) parameter estimator and the robust standard error estimator; in Mplus, this procedure is called as maximum likelihood estimation with robust standard error correction (MLR), which is useful for non-normal and non-independent observations (Muthén and Satorra, 1995). Different from using the inverse of information matrix as the sampling variance estimate with normal distribution assumption, an asymptotically consistent estimate of covariance matrix is derived directly from observations by including a scaling matrix in between two copies of the Hessian matrix and then is used to compute the robust estimate of sampling variance, which is the square of standard error (Huber, 1967; White, 1980; Hardin and Hilbe, 2007). The chi-square test statistic reported using MLR is asymptotically equivalent to Yuan-Bentler T2^*^ test statistic (Muthén and Muthén, 2012). We compared each model performance in simulation convergence rate (CR), model-fit test statistic and fit indices, Information Criteria (i.e., AIC, BIC and adjusted BIC), and the estimates of between/within-level factor loadings, scale reliabilities, residual variance and mean structure estimates as well as their 95% coverage rate and empirical power. Level-specific scale reliability was calculated based on Geldhof et al. (2013) using Equation (9) to decompose variance in an item into the individual component and the cluster component.

## Results

### Convergence rate of simulations, model fit test statistic, fit indices and information criteria

**Figure 3:**
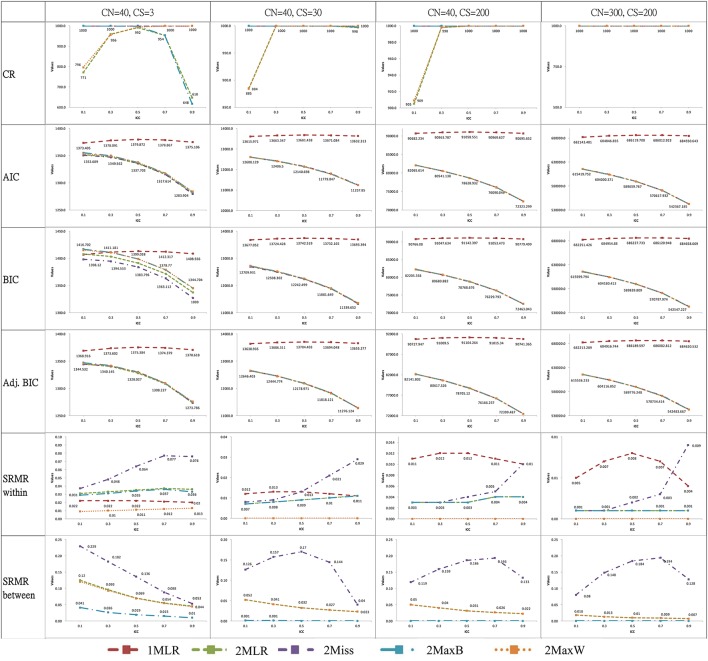
Plots of selected analytical outputs of ICC against fit statistics across different modeling strategies. CN, Cluster number; CS, Cluster size; CR, Convergence rate of simulations; ICC, Intraclass correlation. 1MLR, the one-level design-based model; 2MLR, the two-level model-based model and the true model; 2MaxB, the two-level maximum model with saturated model in between level and true model in within level; 2MaxW, the two-level maximum model with true model in between level and saturated model in within level; 2Miss, the miss-specified two-level model by constraining the factor loading estimates of the between and within levels to be the same.

For ease of illustration, we selected the results of simulation conditions with the smallest cluster number (CN = 40 with CS = 3, 30, 200) and the largest combination of sample size (CN = 300 with CS = 200) in Figure 3. When CN larger than 40, the five modeling techniques achieved convergent results across different ICC conditions. Nevertheless, with a cluster of 40, the convergence ratio varied with ICC values: 2Miss and 1MLR reached 100% convergence for all ICCs, but 2MLR, 2MaxB and 2MaxW had 9.5~38.2% non-convergent simulation results when ICC was smaller than 0.3 or larger than 0.7. For instance, in the smallest case of CN(CS) = 40(3), the CR pattern of the five modeling techniques differed with ICC values: 1MLR and 2Miss reached perfect convergence in all ICC conditions; the CR for 2MaxW exhibited a quadratic pattern, which increased with the increase of ICC and leveled off and reached 100% when ICC ≥0.5 while 2MaxB demonstrated a reversed pattern. 2MLR had a downward-U quadratic pattern of CRs verse ICCs with the peak at ICC = 0.5. According to the error message, the non-convergent result of 2-level models was mostly due to the non-positive definite first-order derivative product matrix for the insufficient portion of variance in the within or between level, especially in the smaller sample size conditions.

All models yielded significant Chi-square exact test results but adequate CFIs, RMSEAs, and SRMR-W values (e.g., *CFI* > 0.90, *RMSEA* < 0.08 and *SRMR* < 0.08, Hu and Bentler, 1999) in all simulation conditions. However, for SRMR-Bs, 2Miss consistently demonstrated badness of fit across most of simulation conditions. Particularly, the SRMS-Bs of the 2Miss showed a quadratic pattern with downward-U shape and peaked between 0.5 and 0.7 for models with a sample size equal or greater than CN(CS) = 40(30). The result suggested that the 2Miss showed lack of fit to the multilevel measurement dataset with level-varying parameters.

Across all simulation conditions, four 2-level models consistently generated smaller AIC and adj. BIC than the 1MLR. The average difference of AIC and adj. BIC between 2-level models and 1MLR were larger than 20 for all the simulation cases even for the smallest sample size conditions (e.g., for [CN(CS), ICC] = [40(3), 0.1], AIC_1MLR_ = 1,373.41 vs. AIC_2MaxB_ = 1,355.38, and adj. BIC_1MLR_ = 1,368.92 vs. adj. BIC_2MaxB_ = 1,347.15). AIC and adj. BIC indices preferred model-based approaches over design-based approaches across all simulation settings. BIC could distinguish the 2-level models from 1MLR in most of simulation conditions, but not for the conditions with the smallest sample CN(CS) = 40(3) at ICC < 0.3.

### Estimation of fixed effects

The parameter estimates of [CN(CS), ICC] = [300(200), 0.3], [40(30), 0.3] and [40(3), 0.3] were summarized in Tables 1–3. Besides, the relative and absolute bias values of estimated factor loadings of CPCSA and CPCAC were tabulated in Table 4,^2^.

**Table 1:** Simulated unstandardized results^a^ of SEM techniques on synthetic harter's competence dataset for [CN(CS), ICC] = [300(200), 0.3].

		**Population settings**		**2MLR**				**1MLR**				**2MaxB**				**2Miss**				**2MaxW**			
Convergence rate		----		1.00				1.00				1.00				1.00				1.00			
Chi-square (df)		----		4.113 (4)				6.490 (2)				2.057(2)				80.343 (7)				2.082 (2)			
CFI		----		1.00				0.998				1.00				0.998				1.00			
RMSEA		----		0.001				0.005				0.001				0.013				0.001			
SRMR(SRMR_B)		---- (----)		0.001 (0.013)				0.007				0.001(<0.001)				0.001 (0.148)				<0.001 (0.013)			
AIC		----		604,009.427				684,769.911				604,011.412				604,079.363				604,011.391			
BIC		----		604,189.469				684,877.936				604,209.458				604,232.398				604,209.437			
ABIC		----		604,125.909				684,839.800				604,139.542				604,178.372				604,139.520			
		**Population**		**Est**.	**SE**	**95%**	**Sig**.	**Est**.	**SE**	**95%**	**Sig**	**Est**.	**SE**	**95%**	**Sig**	**Est**.	**SE**	**95%**	**Sig**	**Est**.	**SE**	**95%**	**Sig**
**WITHIN-LEVEL**																							
	W_CPC by																						
	CPCSC	1.000		1.000	–	–	–	1.000	–	–	–	1.000	–	–	–	1.000	–	–	–				
	CPCSA	0.450		0.450	0.005	0.944	1.00	0.534	0.021	0.023	1.00	0.450	0.005	0.944	1.00	0.451	0.005	0.000	1.00				
	CPCAC	0.920	^**^	0.920	0.008	0.945	1.00	0.791	0.033	0.036	1.00	0.920	0.008	0.945	1.00	0.918	0.008	0.000	1.00				
	CPCSW	0.360		0.360	0.005	0.953	1.00	0.428	0.020	0.061	1.00	0.360	0.005	0.953	1.00	0.361	0.005	0.000	1.00				
	Ψ*_*W*_*CPC*_*	0.700	^**^	0.700	0.009	0.955	1.00	1.036	0.052	0.000	1.00	0.700	0.009	0.955	1.00	0.701	0.008	0.959	1.00				
	**Residual Variance**																						
	*CPCSC*	0.500	^**^	0.500	0.006	0.951	1.00	0.662	0.035	0.003	1.00	0.500	0.006	0.951	1.00	0.499	0.006	0.947	1.00				
	*CPCSA*	0.500	^**^	0.500	0.003	0.943	1.00	0.729	0.021	0.000	1.00	0.500	0.003	0.943	1.00	0.500	0.003	0.943	1.00				
	*CPCAC*	0.500	^**^	0.500	0.006	0.951	1.00	0.753	0.028	0.000	1.00	0.500	0.006	0.951	1.00	0.501	0.006	0.947	1.00				
	*CPCSW*	0.500	^**^	0.500	0.003	0.948	1.00	0.716	0.019	0.000	1.00	0.500	0.003	0.948	1.00	0.500	0.003	0.950	1.00				
**BETWEEN-LEVEL**																							
	B_CPC by																						
	CPCSC	1.000		1.000	–	–	–									1.000	–	–	–	1.000	–	–	–
	CPCSA	0.780	^**^	0.785	0.084	0.949	1.00									0.451	0.005	0.000	1.00	0.785	0.084	0.949	1.00
	CPCAC	0.600	^**^	0.603	0.071	0.955	1.00									0.918	0.008	0.000	1.00	0.603	0.071	0.955	1.00
	CPCSW	0.620	^**^	0.622	0.073	0.949	1.00									0.361	0.005	0.000	1.00	0.622	0.073	0.949	1.00
	Ψ*_*B*_*CPC*_*	0.300		0.301	0.045	0.941	1.00									0.255	0.029	0.630	1.00	0.301	0.045	0.941	1.00
	**Residual variance**																						
	*CPCSC*	0.200	^**^	0.197	0.030	0.939	0.999									0.239	0.031	0.777	1.00	0.197	0.030	0.939	1.00
	*CPCSA*	0.200	^**^	0.199	0.023	0.945	1.00									0.273	0.024	0.102	1.00	0.199	0.023	0.945	1.00
	*CPCAC*	0.200	^**^	0.198	0.020	0.935	1.00									0.153	0.022	0.415	1.00	0.198	0.020	0.935	1.00
	*CPCSW*	0.200	^**^	0.199	0.020	0.937	1.00									0.244	0.021	0.459	1.00	0.199	0.020	0.937	1.00
**INTERCEPT/MEAN**																							
	CPCSC	2.896	^**^	2.898	0.041	0.937	1.00	2.898	0.041	0.937	1.00	2.898	0.041	0.937	1.00	2.898	0.041	0.937	1.00	2.898	0.041	0.937	1.00
	CPCSA	2.856	^**^	2.857	0.036	0.957	1.00	2.857	0.036	0.957	1.00	2.857	0.036	0.957	1.00	2.857	0.036	0.957	1.00	2.857	0.036	0.957	1.00
	CPCAC	2.860	^**^	2.862	0.032	0.943	1.00	2.862	0.032	0.943	1.00	2.862	0.032	0.043	1.00	2.862	0.032	0.943	1.00	2.862	0.032	0.943	1.00
	CPCSW	3.268	^**^	3.268	0.033	0.946	1.00	3.268	0.033	0.946	1.00	3.268	0.033	0.946	1.00	3.268	0.033	0.946	1.00	3.268	0.033	0.946	1.00

Ψ_B_CPC_ and Ψ_W_CPC_ is the between-/within-level factor variance. The normal font indicates the fixed effect and intercept estimate; the italic indicates the random effect estimate. Est, estimate; SE, standard error; 95%, 95% confidence interval coverage rate; Sig., empirical power.

** *p < 0.05*.

a *The standardized result can be requested from the author*.

**Table 2:** Simulated unstandardized results^a^ of SEM techniques on synthetic harter's competence dataset for [CN(CS), ICC] = [40(30), 0.3].

		**Population settings**		**2MLR**				**1MLR**				**2MaxB**				**2Miss**				**2MaxW**			
Convergence rate		----		0.999				1.00				1.00				1.00				0.998			
Chi-square (df)		----		4.935 (4)				3.041 (2)				2.041 (2)				16.832 (7)				3.280 (2)			
CFI		----		0.998				0.995				0.999				0.988				0.998			
RMSEA		----		0.011				0.015				0.010				0.015				0.015			
SRMR(SRMR_B)		---- (----)		0.008 (0.043)				0.013				0.008 (0.001)				0.009 (0.158)				<0.001 (0.043)			
AIC		----		12,406.494				13,661.328				12,408.438				12,412.245				12,408.726			
BIC		----		12,508.295				13,722.409				12,520.42				12,498.776				12,520.708			
ABIC		----		12,444.768				13,684.292				12,450.54				12,444.777				12,450.828			
		**Population**		**Est**.	**SE**	**95%**	**Sig**.	**Est**.	**SE**	**95%**	**Sig**	**Est**.	**SE**	**95%**	**Sig**	**Est**.	**SE**	**95%**	**Sig**	**Est**.	**SE**	**95%**	**Sig**
**WITHIN-LEVEL**																							
	W_CPC by																						
	CPCSC	1.000		1.000	–	–	–	1.000	–	–	–	1.000	–	–	–	1.000	–	–	–				
	CPCSA	0.450		0.451	0.034	0.936	1.00	0.533	0.064	0.757	1.00	0.450	0.034	0.937	1.00	0.457	0.034	0.937	1.00				
	CPCAC	0.920	^**^	0.922	0.059	0.929	1.00	0.795	0.097	0.680	1.00	0.922	0.059	0.929	1.00	0.907	0.058	0.918	1.00				
	CPCSW	0.360		0.359	0.032	0.949	1.00	0.426	0.060	0.816	1.00	0.359	0.032	0.950	1.00	0.365	0.032	0.948	1.00				
	Ψ_*W*_*CPC*_	0.700	^**^	0.700	0.602	0.932	1.00	1.041	0.156	0.421	1.00	0.703	0.060	0.932	1.00	0.708	0.060	0.931	1.00				
	**Residual Variance**																						
	*CPCSC*	0.500	^**^	0.500	0.045	0.941	1.00	0.653	0.107	0.679	1.00	0.498	0.045	0.941	1.00	0.494	0.045	0.933	1.00				
	*CPCSA*	0.500	^**^	0.500	0.022	0.943	1.00	0.719	0.061	0.021	1.00	0.499	0.022	0.943	1.00	0.497	0.023	0.936	1.00				
	*CPCAC*	0.500	^**^	0.500	0.040	0.943	1.00	0.743	0.083	0.156	1.00	0.498	0.040	0.942	1.00	0.507	0.039	0.931	1.00				
	*CPCSW*	0.500	^**^	0.500	0.022	0.937	1.00	0.708	0.057	0.009	1.00	0.499	0.022	0.937	1.00	0.498	0.022	0.935	1.00				
**BETWEEN-LEVEL**																							
	B_CPC by																						
	CPCSC	1.000		1.000	–	–	–									1.000	–	–	–	1.000	–	–	–
	CPCSA	0.780	^**^	1.042	0.421	0.925	0.874									0.457	0.034	0.000	1.00	0.928	0.542	0.926	0.876
	CPCAC	0.600	^**^	0.621	0.244	0.933	0.801									0.907	0.058	0.000	1.00	0.621	0.245	0.935	0.801
	CPCSW	0.620	^**^	0.653	0.262	0.928	0.819									0.365	0.032	0.000	1.00	0.651	0.262	0.929	0.820
	Ψ_*B*_*CPC*_	0.300		0.348	0.494	0.905	0.664									0.261	0.091	0.817	0.935	0.314	0.147	0.907	0.665
	**Residual Variance**																						
	*CPCSC*	0.200	^**^	0.145	0.455	0.935	0.598									0.230	0.088	0.939	1.00	0.179	0.108	0.937	0.597
	*CPCSA*	0.200	^**^	0.163	0.102	0.907	0.799									0.261	0.064	0.893	1.00	0.172	0.120	0.906	0.801
	*CPCAC*	0.200	^**^	0.186	0.056	0.889	0.950									0.154	0.064	0.773	1.00	0.187	0.056	0.888	0.950
	*CPCSW*	0.200	^**^	0.186	0.058	0.889	0.942									0.236	0.057	0.927	1.00	0.186	0.058	0.890	0.940
**INTERCEPT/MEAN**																							
	CPCSC	2.896	^**^	2.896	0.115	0.945	1.00	2.896	0.116	0.947	1.00	2.896	0.115	0.945	1.00	2.896	0.115	0.945	1.00	2.896	0.115	0.945	1.00
	CPCSA	2.856	^**^	2.852	0.099	0.936	1.00	2.852	0.100	0.939	1.00	2.852	0.099	0.936	1.00	2.852	0.099	0.936	1.00	2.852	0.099	0.936	1.00
	CPCAC	2.860	^**^	2.862	0.091	0.933	1.00	2.872	0.092	0.935	1.00	2.862	0.091	0.933	1.00	2.862	0.091	0.933	1.00	2.862	0.091	0.934	1.00
	CPCSW	3.268	^**^	3.270	0.090	0.947	1.00	3.270	0.091	0.948	1.00	3.270	0.090	0.947	1.00	3.270	0.090	0.947	1.00	3.270	0.090	0.947	1.00

*Ψ_B_CPC_ and Ψ_W_CPC_ is the between-/within-level factor variance. The normal font indicates the fixed effect and intercept estimate; the italic indicates the random effect estimate. Est, estimate; SE, standard error; 95%, 95% confidence interval coverage rate; Sig., empirical power*.

** *p < 0.05*.

a *The standardized result can be requested from the author*.

**Table 3:** Simulated unstandardized results^a^ of SEM techniques on synthetic harter's competence dataset for [CN(CS), ICC] = [40(3), 0.3].

		**Population settings**		**2MLR**				**1MLR**				**2MaxB**				**2Miss**				**2MaxW**			
Convergence rate		----		0.786				1.000				0.820				0.879				0.782			
Chi-square (df)		----		8.674 (4)				2.680 (2)				27.99 (2)				12.63 (7)				8.111 (2)			
CFI		----		0.959				0.982				0.968				0.941				0.963			
RMSEA		----		0.056				0.042				0.078				0.063				0.070			
SRMR(SRMR_B)		---- (----)		0.033 (0.085)				0.023				0.032 (0.025)				0.050 (0.173)				0.010 (0.082)			
AIC		----		1,347.312				1,368.669				1,347.010				1,347.254				1,349.315			
BIC		----		1,403.062				1,402.119				1,408.335				1,394.641				1,410.640			
ABIC		----		1,339.831				1,364.181				1,338.781				1,340.895				1,341.086			
		**Population**		**Est**.	**SE**	**95%**	**Sig**.	**Est**.	**SE**	**95%**	**Sig**	**Est**.	**SE**	**95%**	**Sig**	**Est**.	**SE**	**95%**	**Sig**	**Est**.	**SE**	**95%**	**Sig**
**WITHIN-LEVEL**																							
	W_CPC by																						
	CPCSC	1.000		1.000	–	–	–	1.000	–	–	–	1.000	–	–	–	1.000	–	–	–				
	CPCSA	0.450		0.453	0.146	0.944	0.808	0.607	0.154	0.891	0.988	0.474	0.147	0.954	0.829	0.509	0.123	0.941	0.991				
	CPCAC	0.920	^**^	0.952	0.287	0.949	0.951	0.910	0.215	0.965	0.998	0.988	0.286	0.980	0.965	0.839	0.179	0.863	0.996				
	CPCSW	0.360		0.358	0.137	0.944	0.796	0.484	0.140	0.909	0.963	0.374	0.140	0.951	0.819	0.407	0.112	0.939	0.960				
	Ψ_*W*_*CPC*_	0.700	^**^	0.723	0.265	0.938	0.881	1.061	0.246	0.994	0.992	0.654	0.232	0.935	0.904	0.720	0.214	0.925	0.973				
	**Residual Variance**																						
	*CPCSC*	0.500	^**^	0.486	0.206	0.964	0.732	0.739	0.191	0.781	0.983	0.514	0.183	0.962	0.807	0.479	0.165	0.937	0.829				
	*CPCSA*	0.500	^**^	0.491	0.085	0.924	0.999	0.693	0.115	0.623	1.00	0.488	0.085	0.919	1.00	0.485	0.086	0.915	1.00				
	*CPCAC*	0.500	^**^	0.470	0.174	0.947	0.796	0.683	0.165	0.755	0.967	0.456	0.169	0.953	0.787	0.542	0.139	0.927	0.952				
	*CPCSW*	0.500	^**^	0.487	0.081	0.922	1.00	0.687	0.105	0.588	1.00	0.484	0.081	0.913	0.999	0.484	0.081	0.916	1.00				
**BETWEEN-LEVEL**																							
	B_CPC by																						
	CPCSC	1.000		1.000	–	–	–									1.000	–	–	–	1.000	–	–	–
	CPCSA	0.780	^**^	0.930	0.812	0.926	0.409									0.509	0.123	0.356	0.991	1.128	0.830	0.908	0.419
	CPCAC	0.600	^**^	0.681	0.582	0.964	0.428									0.839	0.179	0.784	0.996	0.667	0.536	0.965	0.438
	CPCSW	0.620	^**^	0.721	0.747	0.945	0.363									0.407	0.112	0.457	0.960	0.700	0.594	0.941	0.375
	Ψ_*B*_*CPC*_	0.300		0.337	0.289	0.935	0.200									0.338	0.198	0.950	0.340	0.342	0.288	0.934	0.195
	**Residual Variance**																						
	*CPCSC*	0.200	^**^	0.158	0.210	0.955	0.183									0.165	0.146	0.909	0.173	0.156	0.214	0.955	0.190
	*CPCSA*	0.200	^**^	0.140	0.189	0.938	0.302									0.233	0.101	0.930	0.647	0.109	0.213	0.934	0.299
	*CPCAC*	0.200	^**^	0.162	0.140	0.930	0.268									0.164	0.116	0.891	0.251	0.164	0.135	0.932	0.272
	*CPCSW*	0.200	^**^	0.154	0.166	0.932	0.354									0.215	0.092	0.934	0.678	0.158	0.137	0.928	0.364
**INTERCEPT/MEAN**																							
	CPCSC	2.896	^**^	2.903	0.149	0.943	1.00	2.909	0.140	0.932	1.00	2.903	0.147	0.933	1.00	2.903	0.149	0.939	1.00	2.902	0.149	0.941	1.00
	CPCSA	2.856	^**^	2.862	0.121	0.938	1.00	2.863	0.120	0.957	1.00	2.861	0.120	0.948	1.00	2.862	0.120	0.942	1.00	2.862	0.121	0.938	1.00
	CPCAC	2.860	^**^	2.863	0.129	0.946	1.00	2.867	0.127	0.948	1.00	2.863	0.127	0.942	1.00	2.864	0.129	0.944	1.00	2.863	0.129	0.943	1.00
	CPCSW	3.268	^**^	3.264	0.112	0.941	1.00	3.267	0.112	0.938	1.00	3.267	0.111	0.934	1.00	3.268	0.111	0.936	1.00	3.264	0.112	0.938	1.00

*Ψ_B_CPC_ and Ψ_W_CPC_ is the between-/within-level factor variance. The normal font indicates the fixed effect and intercept estimate; the italic indicates the random effect estimate. Est, estimate; SE, standard error; 95%, 95% confidence interval coverage rate; Sig., empirical power*.

** *p < 0.05*.

a *The standardized result can be requested from the author*.

**Table 4:** The relative bias and absolute bias of factor loading estimates from five SEM modeling techniques for ICC = 0.3.

**CN(CS)**	**Model**	**Within Level**				**Between Level**			
		**CPCSA**		**CPCAC**		**CPCSA**		**CPCAC**	
		**Bias (%)**	**Abs(Bias) (%)**	**Bias (%)**	**Abs(Bias) (%)**	**Bias (%)**	**Abs(Bias) (%)**	**Bias (%)**	**Abs(Bias) (%)**
40(3)	2MLR	0.72	24.58	3.52	21.88	19.18	53.69	13.41	52.32
	2MaxB	5.21	24.24	7.35	20.57				
	2MaxW					19.55	54.57	11.11	49.29
	2Miss	13.13	23.66	−8.84	17.47	−34.74	35.32	39.78	41.25
	1MLR	20.79	28.16	−12.02	19.03				
	1MLR^*^	−0.99	19.13	−1.77	17.03				
40(30)	2MLR	0.23	6.17	0.19	5.27	14.63	39.07	3.55	30.88
	2MaxB	0.22	6.17	0.19	5.27				
	2MaxW					15.48	39.94	3.56	30.87
	2Miss	1.59	6.23	−1.43	5.37	−41.39	41.39	51.14	51.14
	1MLR	18.49	19.74	−13.55	14.92				
	1MLR^*^	2.48	12.03	−2.03	7.75				
300(200)	2MLR	−0.02	0.89	0.02	0.72	0.62	8.76	0.57	9.41
	2MaxB	−0.02	0.89	0.02	0.72				
	2MaxW					0.62	8.76	0.57	9.41
	2Miss	0.19	0.90	−0.22	0.75	−42.20	42.20	52.99	52.99
	1MLR	18.67	18.67	−14.01	14.01				
	1MLR^*^	−2.73	3.87	−3.92	4.63				

Bias: relative bias = $\frac{Estimate-Parameter}{Parameter}$ ; Abs(Bias) = $\left|\frac{Estimate\;-Parameter}{Parameter}\right|$. The parameter value of 2-level models and 1MLR in the within level: λ_CPCSA_ = 0.45, λ_CPCAC_ = 0.92 and in the between level: λ_CPCSA_ = 0.78, λ_CPCAC_ = 0.60 of population two-level model. 1MLR

* *presents the bias measures with respect to its true conflated parameter value from Equation (8): λ_CPCSA_ = 0.549, λ_CPCAC_ = 0.824*.

CPCSC was the maker variable so its factor loading would constantly be fixed at one for all the analytical models. CPCSW and CPCSA had the same pattern of bias; therefore, we presented the result of for CPCSA and CPCAC only. Relative bias (RB) is calculated as the value of parameter estimate minus the population value divided by the population value. RB quantifies the degree of deviation of the parameter estimate relative to the population value. A zero value of RB reflects an unbiased estimate of the parameter. A negative value indicates an underestimation of the parameter; on the other hand, a positive value indicates an overestimation of the parameter. According to Flora and Curran (2004), the value of RB less than 5% is considered as trivial, between 5 and 10% as moderate, and greater than 10% as substantial. Absolute bias (AB) is the absolute value of RB, which will always be positive and cumulated to reflect the total amount of bias. Across simulation settings, 2MLR, 2MaxB, and 2MaxW models tended to generate factor loading estimates consistent with the population values in respective levels. The empirical results were consistent with the mathematical derivations [e.g., Equation (4), (5.1) and (5.2)]. Generally, as shown in Table 4, ABs were larger than their RB counterparts in smaller CN and CS, but as CN and CS increased, the discrepancy between RB and AB were smaller. The RBs and ABs of the parameter estimates were also getting smaller when sample size increased for 2-level models, except that 2Miss consistently generated biased between-level loading estimates across all sample size settings.

On the other hand, 1MLR and 2Miss tended to generate conflated estimates for the factor loadings, consistent with Equation (7). Take the condition of the smallest sample size as example [CN(CS), ICC] = [40(3), 0.3], compared with the within-level fixed effects in the population model, substantial relative bias was found in the factor loading estimates of 1MLR and 2Miss ranging from −12.02 to 20.79%. In contrast, negligible relative bias of factor loading estimates was found in the 2MLR and 2MaxB models ranging from 0.72 to 7.35% (e.g., $\lambda_{CPCSA,\;W\text{\_}CPC}^{True\;model}=0.450$, ${\hat{\lambda }}_{CPCSA,W\text{\_}CPC}^{2MLR}=0.453\;$and ${\hat{\lambda }}_{CPCSA,W\text{\_}CPC}^{2MaxB}=0.474\;$vs. ${\hat{\lambda }}_{CPCSA,CPC}^{1MLR}=0.607$, and ${\hat{\lambda }}_{CPCSA,W\text{\_}CPC}^{2Miss}=0.509$). We also compared the factor loading estimates of 1MLR with its theoretical conflated values (obtained from Equation (8) with ICC = 0.3, e.g., $\lambda_{CPCSA,CPC}^{1MLR}=0.549$) and presented the biases in Table 4 at the row of 1MLR^*^. 1MLR generated negligible biases which grew larger as sample size increased (e.g., the relative bias ranged from −0.99 to −3.92%). Compared with the between-level fixed effects in the population model, the 2MLR and 2MaxW models yielded considerable relative and absolute biases at CN = 40 (the relative bias ranged from 3.55 to 19.55%; the absolute bias ranged from 54.57 to 30.87%).

**Figure 4:**
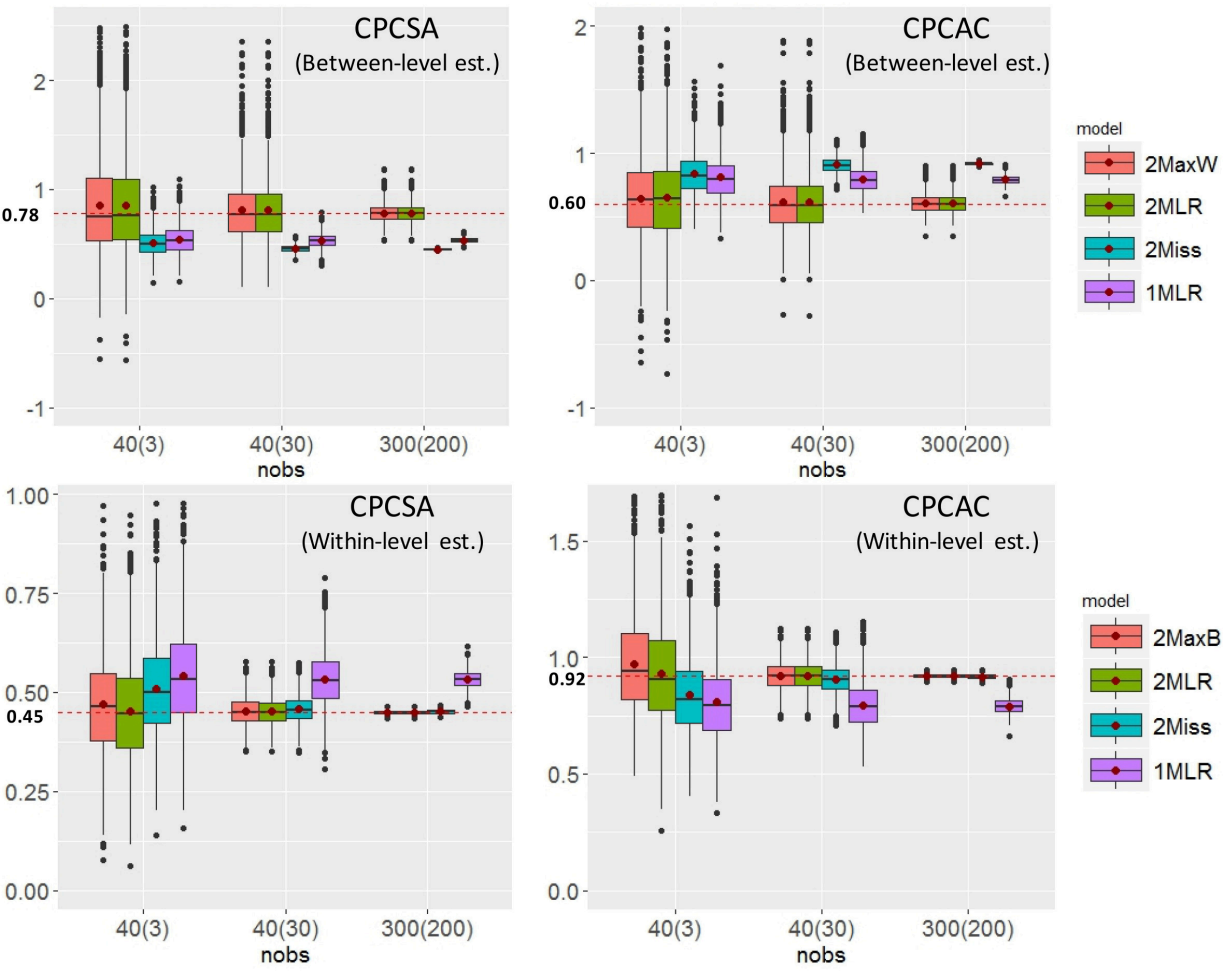
The Boxplots of selected factor loading estimates vs. sample size conditions. The red dots in the boxes indicate the means of factor loading estimates. The red dashed lines indicates the parameter settings in respective levels.

To further investigate the relationship between factor loading estimates and sample sizes (e.g., CN × CS), we tabulated the between- and within-level $\hat{\lambda }$ of CPCSA and CPCAC in boxplots for ICC = 0.3 in Figure 4. The dispersion of the parameter estimates of the five models decreased as the sample size increased. When sample size was small, the dispersion of 2MLR was larger than 2MaxB/2MaxW. Across all cluster number and cluster size combinations, the 2MLR and the 2MaxB/2MaxW had consistent median estimates to their parameters. However, the 1MLR models generated conflated parameter estimates which would regress to the weighted means of the true factor loadings from the between-and within-level models (The true value of within-level $\lambda_{CPCAC,\;W\text{\_}CPC}^{True\;model}=0.920$, between-level $\lambda_{CPCAC,\;B\text{\_}CPC}^{True\;model}=0.600$, and the conflated parameter $\lambda_{CPCSA,CPC}^{1MLR}=0.824$, vs. the estimate of ${\hat{\lambda }}_{CPCAC,\;CPC}^{1MLR}=0.791$; $\lambda_{CPCSA,\;W\text{\_}CPC}^{True\;model}=0.450$, $\lambda_{CPCSA,\;B\text{\_}CPC}^{True\;model}=0.780$, and $\lambda_{CPCSA,CPC}^{1MLR}=0.549$ vs. ${\hat{\lambda }}_{CPCSA,\;CPC}^{1MLR}=0.534$). Different from 1MLR, the 2Miss models had consistent and efficient factor loading estimates as those produced by the 2MLR and 2MaxB models when sample size was greater than 1,200 [i.e., CN(CS) = 40(30)] in the within-level models; whereas, the 2Miss models generated biased parameter estimates across all sample size conditions in the between-level level.

### The conflated factor loading estimates in design-based models as ICC changes

**Figure 5:**
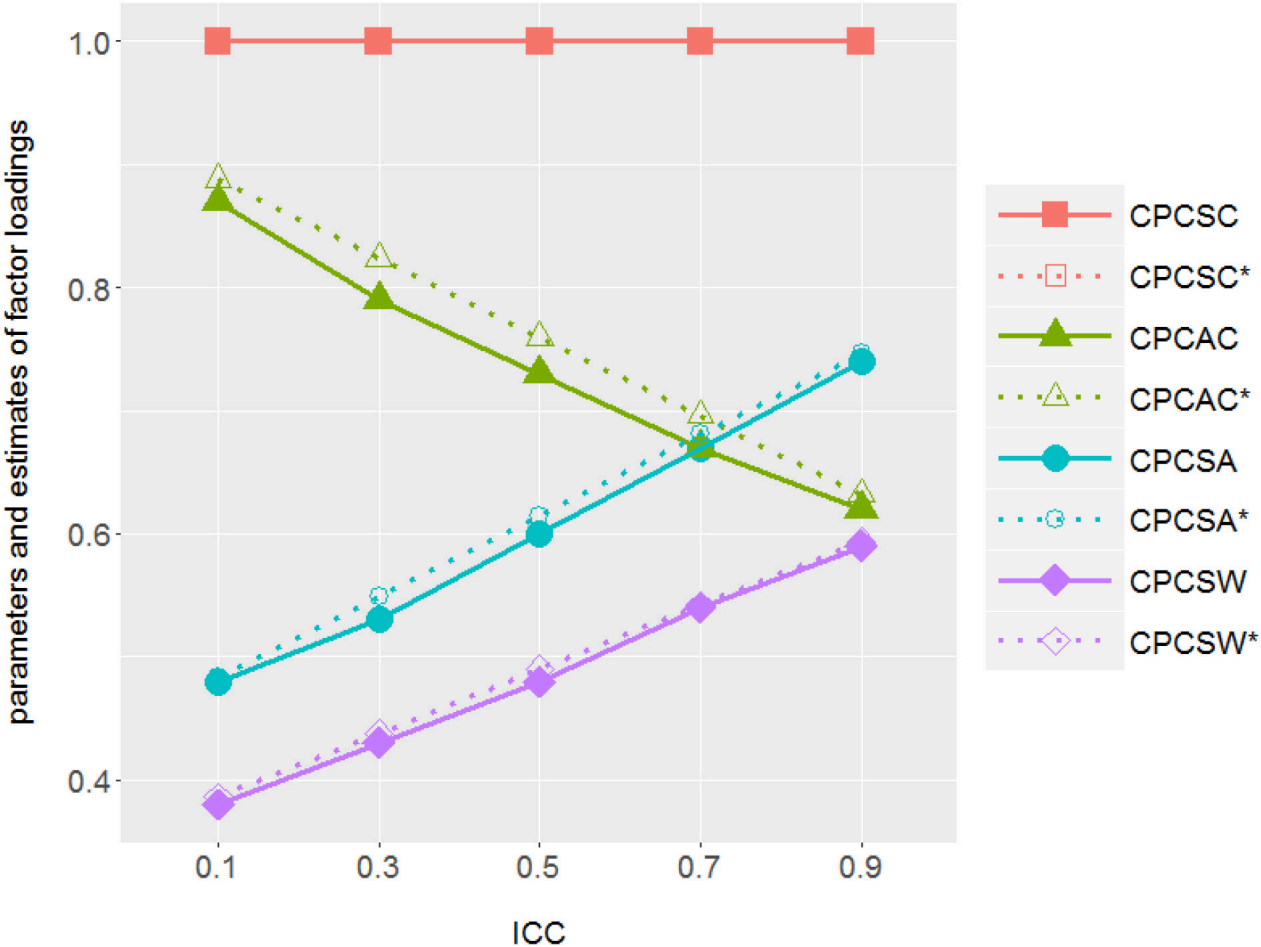
ICCs vs. parameter estimates from the simulations and those from the mathematical derivations of the design-based approach: As ICC increases, design-based approach tends to generate factor loading estimates which are closer to its between-level counterpart and deviate from its within-level values in the true model. There is one factor in both within and between levels with factor variance **Ψ**_*Within*−*level*_ = (**1**−**ICC**)·**Ψ**_*total*_ and **Ψ**_*Between*−*level*_ = **ICC**·**Ψ**_*total*_ with **Ψ**_*total*_ **= Ψ**_*Between*−*level*_ **+ Ψ**_*Within*−*level*_ **=** 1. Solid line illustrates the factor loading estimates of design-based approach (1MLR) from simulations; dotted line illustrates the theoretical parameter values of design-based approach. CPCSC is the marker variable. The true value of CPCAC $\lambda_{CPCAC,\;W\_CPC}^{True\;model}\;=\;0.920$, $\lambda_{CPCAC,\;B\_CPC}^{True\;model}=0.600$; CPCSA $\lambda_{CPCSA,\;W\_CPC}^{True\;model}\;=\;0.450$ and $\lambda_{CPCSA,\;B\_CPC}^{True\;model}=0.780$; CPCSA $\lambda_{CPCSW,\;W\_CPC}^{True\;model}\;=\;0.360$ and $\lambda_{CPCSW,\;B\_CPC}^{True\;model}=0.620$.

To probe into the consequence of applying design-based approach on complex survey data, we plotted the estimates (solid lines) of factor loadings from simulations and those (dash lines) from mathematical derivation (see Equation 8) against different ICC values in Figure 5. As we expected from the mathematical derivation, the factor loading estimates of the design-based model approached the true between-level values as ICCs increased. Even though they were supposed to reflect the within-level information, the estimates got conflated across all simulated ICCs, except for ICC = 0.

### Estimation of random effects

In terms of factor variance, the four 2-level models yielded consistent random effect estimates (e.g., for [40(3), 0.3], in the between level: ${\hat{\text{Ψ}}}_{B\text{\_}CPC}^{2MLR}=0.337,\;{\hat{\text{Ψ}}}_{B\text{\_}CPC}^{2MaxW}=0.342\text{ and }{\hat{\text{Ψ}}}_{B\text{\_}CPC}^{2Miss}=0.338$; in the within level: ${\hat{\text{Ψ}}}_{W\text{\_}CPC}^{2MLR}=0.723,\;{\hat{\text{Ψ}}}_{W\text{\_}CPC}^{2MaxB}=0.654$, ${\hat{\text{Ψ}}}_{W\text{\_}CPC}^{2Miss}=0.720$). The performance of the 1MLR, however, was not as consistent as that of the three 2-level models in estimating the random effects. Specifically, the factor variance estimate of 1MLR equaled 1.061, which was roughly the sum of the population between- and within-level factor variance values as shown in Equation (6). The substantial relative bias reached 51.57%. The 1MLR also yielded the same overall estimates for the residual variances (i.e., residuals of Equation 7), while the three 2-level models had fair within-level residual variance estimate (e.g., ${\hat{\theta }}_{CPCSW}^{1MLR}=0.687$ vs. $\theta_{CPCSW,\;Within-level}^{True\;model}=0.500$, ${\hat{\theta }}_{CPCSW,\;Within-level}^{2MLR}=0.487$, ${\hat{\theta }}_{CPCSW,\;Within-level}^{2MaxB}=0.484$ and ${\hat{\theta }}_{CPCSW,\;Within-level}^{2Miss}=0.484$).

### Mean structures

As for the mean structure, all examined models yielded consistent mean/intercept estimates with conformable statistical inferences as shown in Tables 1–3.

### The 95% confidence interval coverage rate and empirical power of estimates

With the conflated parameter estimate of fixed and random effect, the 95% confidence interval coverage rate^3^ (95%) of 1MLR and 2Miss tended to be much smaller than its nominal level. In terms of empirical power^4^ (Sig.), all the empirical power for the three factor loading estimates were equal to or close to 1 in the 1MLR and 2Miss (e.g., for [40(3), 0.3], ${\hat{\lambda }}_{CPCSA,CPC}^{1MLR}=0.659$, 95% = 0.891, Sig = 0.988 in Table 3). In contrast, in the 2MLR and 2MaxB models, the empirical power of ${\hat{\lambda }}_{CPCSA,W\text{\_}CPC}$ and ${\hat{\lambda }}_{CPCSW,W\text{\_}CPC}$ were both close to 0.8 (e.g., ${\hat{\lambda }}_{CPCSW,W\text{\_}CPC}^{2MLR}=0.358$, 95% = 0.944, Sig = 0.796; ${\hat{\lambda }}_{CPCSW,W\text{\_}CPC}^{2MaxB}$, 95% = 0.951, Sig = 0.819). In the true model, these two factor loadings were considered as non-zero and smaller effects without statistical significance at small sample size. With the small sample size setting in the simulation, this kind of smaller effects were set to have less empirical rate of significant estimates over total replications than the nominal level of 0.8 (Eng, 2003). Results of the 2MLR and 2MaxB were consistent with the population model, in which only the empirical power for the factor loading of individual-level athletic competence (CPCAC) far more than 0.8 but not those for social acceptance (CPCSA) and self-worth (CPCSW).

### Variance explained^5^ and scale reliability of indicators

Taking [CN(CS), ICC] = [40(3), 0.3] as an example shown in Table 5, 1MLR tended to generate inflated *R*^2^ measure, especially for the indicators with smaller within-level factor loadings but larger between-level factor loadings, so did the 2Miss model (e.g., ${\hat{R}}_{CPCSA}^{2,\;1MLR}=0.467$ and, ${\hat{R}}_{CPCSA,\;Within-level}^{2,\;2Miss}=0.278$ vs. ${\hat{R}}_{CPCSA,\;Within-level}^{2,\;2MLR}=0.175$ and ${\hat{R}}_{CPCSA,\;Within-level}^{2,\;2MaxB}=0.176$). As for the between-level, 2MaxW provided consistent ${\hat{R}}^{2}$ as 2MLR but 2Miss generated biased estimate (${\hat{R}}_{CPCSA,\;Between-level}^{2,\;2MaxW}=0.746$ and ${\hat{R}}_{CPCSA,\;Between-level}^{2,\;2MLR}=0.740$ vs. ${\hat{R}}_{CPCSA,\;Between-level}^{2,\;2Miss}=0.623$).

**Table 5:** Values of ICC and *R*^2^ on indicators in the synthetic dataset of harter's competence measures using five SEM modeling techniques for [CN(CS), ICC] = [40(3), 0.3].

			**CPCSC**	**CPCSA**	**CPCAC**	**CPCSW**	**Scale reliabilityρ**
ICC			0.617	0.612	0.352	0.431	---
*R*^2^	2MLR	Within-level	0.506	0.175	0.473	0.124	0.825
		Between-level	0.802	0.740	0.651	0.662	0.930
	1MLR		0.697	0.467	0.482	0.370	0.747
	2MaxB	Within-level	0.503	0.176	0.473	0.124	0.830
	2MaxW	Between-level	0.799	0.746	0.653	0.660	0.926
	2Miss	Within-level	0.468	0.278	0.271	0.195	0.798
		Between-level	0.843	0.623	0.745	0.553	0.915

*CPCSC, Harter perceived scholastic competence; CPCSA, Harter perceived social acceptance; CPCAC, Harter perceived athletic competence; CPCSW, Harter perceived global self-worth*.

As for the scale reliability, the 2MaxB and 2MaxW yielded consistent reliability measures as 2MLR in respective levels, but 1-level MLR and 2-level Miss tended to underestimate the score consistency of indicators (e.g., ${\hat{\rho }}_{Within-level}^{2MaxB}=0.830$ $≅{\hat{\rho }}_{Within-level}^{2MLR}=0.825$; ${\hat{\rho }}_{Between-level}^{2MaxW}=0.926$ $≅{\hat{\rho }}_{Between-level}^{2MLR}=0.930$ vs. ${\hat{\rho }}^{1MLR}=0.747$, ${\hat{\rho }}_{Within-level}^{2Miss}=0.798$ and ${\hat{\rho }}_{Between-level}^{2Miss}=0.915$).

In summary, given the conflated estimates of fixed and random effects, the 1MLR models would provide overestimated variance explained measure and underestimated reliability measure for the indicators. In contrast, the 2MaxB and 2MaxW model generated consistent *R*^2^ and ρ for respective within-level and between-level indicators consistent with those of the 2MLR model across simulation settings.

## Discussion and conclusion

As researchers call for the need to adequately take into account of the multilevel structure of social and behavioral data (Skinner et al., 1997; Lee and Forthofer, 2006), the use of multilevel data modeling techniques will be inevitable. However, multilevel models are not an infallible statistical strategy unless the hypothesized model conforms to the real data structure. In this study, we demonstrated that maximum models are robust analytic methods as to the inequality of higher- and lower-level factor loadings or to detect possibly non-significant trivial items, especially when researchers have limited information about the significance pattern of factor loadings and level-varying measurement structures. The current study focuses on multilevel CFA, which is a generic form of structural equation models; therefore, the study result can be generalized to more complex models.

Specifically, we examined the performance of five proposed SEM techniques on analyzing complex survey data with unequal factor loadings under equal between- and within-level structures. Across different combinations of cluster numbers, cluster sizes and ICC values, all models yield acceptable model-fit information. AIC and adjusted BIC could be utilized to differentiate 1MLR from 2-level models but could not select the best 2-level model. Among 2-level models, 2MLR, 2MaxB and 2MaxW could consistently generate the effective and efficient parameter estimates. On the contrary, the design-based model would not be an appropriate approach on analyzing complex survey data due to its conflated fixed and random effect estimates, inflated standard error estimates, and inconsistent statistical inferences, along with the overestimated variance explained and underestimated reliability measures of the indicators. Below we elaborated on the consequences of using design-based models and miss-specified 2-level models as well as the advantages of our recommended methods in analyzing complex survey measurement data.

### Disadvantages of the design-based approach and mis-specified multilevel models

Using both mathematical derivation and empirical data simulation, we demonstrated that the 1MLR as well as 2Miss yields similar but conflated fixed effect; on the other hand, 2Miss could specify level-specific random components while 1MLR would yield overall random effect estimates. When 1MLR model is used, it truly estimates the combination of variations from different levels in a single-level modeling simultaneously. The parameter estimates got mixed with components from both levels except for ICC = 0 and 1 (as shown in Figure 5). In that case, the consequences were spurious fixed effect estimates with more likely statistical significance and bigger *R*^2^. Moreover, with the overall estimate of residual variance, the design-based approach tended to generate smaller scale reliability estimates.

On the other hand, if the model-based approach is miss-specified, researchers will yield parameter estimates which deviate from the population values in respective levels. In this study, we construct the miss-specified model-based model by constraining the between- and within-level factor loadings to be equal, and the consequence of the analytic results is similar to that of the design-based approach because the design-based approach assumes the between and within level model have not only exactly the same structure (Muthén and Satorra, 1995; Wu and Kwok, 2012), but also the same magnitude of factor loadings.

In regression-like analyses, the design-based approach is reliable to generate consistent statistical inference of parameter estimates by adjusting its standard error considering data dependency (Hardin and Hilbe, 2007); however, in CFA or SEM-based analysis, we demonstrate that the design-based approach on complex survey data cannot guarantee consistent statistical inferences of the result to a specific level with conflated parameter estimates. Design-based approaches are beneficial to take the data dependency into consideration by adjusting the estimate of standard error when the between and within levels have equal structures. However, only when the equality in structures and in population values holds for both levels, the analytic result can be unbiased to specific-level inferences. In most of MCFA or MSEM analyses, the parameter estimates obtained from the design-based approach is a function of between- and within-level population values and the analytic result cannot infer to any level. In the case of children's perceived Harter competence, the four factor loadings of different competence aspects were all statistically significant at the classroom level while only the factor loading of athletic competence was significant at the individual level in early childhood, based on a correctly specified and analyzed result. Nevertheless, as shown in the 1MLR and 2Miss, all four factor loadings were statistically significant which could mislead researchers to conclude that all four competence aspects were important for the *individual* development of the overall perceived competence and to invest their efforts to items (aspects) that are trivial or of little importance for early elementary student's individual competence development.

Under the MCFA framework, we provided evidence to illustrate that design-based approaches yield conflated parameter estimates with multilevel measurement data even under equal level structures as long as the population values at each level are different. In reality, we can hardly know the true model and thus should be more cautious about making inferences with estimates from design-based approaches to represent the lower-level model characteristics.

### Advantages of maximum models

To have consistent and unbiased statistical inferences, methodologists debated over the adequacy of model-based approaches and design-based approaches on analyzing multilevel dataset from complex survey sampling (Snijders and Bosker, 2011). Adding new findings to the literature, first, we demonstrated that the design-based approach is not a robust analytic model for multilevel data under equal level structures with unequal factor loadings. Second, the model-based approach can produce unbiased fixed and random effect estimates as well as their corresponding statistical inferences if and only if the model is correctly specified. Third, most importantly, we suggested that 2MaxB and 2MaxW models are robust and feasible techniques for separating variance components from different levels and for investigating possible higher-level and lower-level structures. Fourth, when the number of clusters in the higher-level sampling units is sufficient (e.g., no less than 40 as shown in simulation), the 2MLR and 2MaxW models can yield consistent and effective estimates of the fixed and random effects. By estimating a saturated between- or within-level model, maximum models enable researchers to focus on examining the lower- or higher-level findings and to obtain consistent statistical inference for findings that researchers are interested in. In the current empirical data simulation, compared to those in the design-based model, variables with smaller factor loadings and smaller *R*^2^ in the within level of the maximum model (e.g., social competence in 2MaxB model) may suggest stronger factor loadings in the between level based on the Equation (7). Researchers in the applied area are encouraged to compare results from maximum models with those from design-based models to investigate possible higher level variation and avoid investing unnecessary efforts on unimportant aspects (i.e., trivial items with smaller amount of factor loadings and variance explained).

### Recommendations for practice and limitation

According to the simulation results, information criteria performed better than model-fit test and fit indices in selecting the optimal analytical models on multilevel measurement data. Researchers can refer to information criteria statistics to determine if their hypothesized models fit the multilevel measurement data adequately. They can start by fitting a 2Miss and a 1MLR. If the information criteria suggested better fit for the 2Miss model (e.g., Δ *AIC* or Δ *adj.BIC* ≥ 20), they should go a step further to perform 2MLR when they have theoretical or empirical evidence, or they could specify 2MaxB or 2MaxW depending on their primary interest in the specific level to ensure consistent and effective estimates of the fixed and random effects. Especially 2MaxB is recommended when the number of between-level sampling units is small (e.g., CN < 40) under the setting of 4 or fewer manifest variables. As a caveat, though AIC and adj. BIC reflected better fit for 2-level models than design-based models across all simulation conditions, they were shown to perform poorly in many contexts (e.g., Preacher and Merkle, 2012). More research can be done to investigate the effectiveness of AIC and BIC in model selection across different parameter settings.

Moreover, in this study, we discuss a multilevel measurement model with a uni-factor structure in both levels; however, if the level structure is misspecified, part of the misspecification would still pass on to the other level and influence the modeling result. Thus, it is possible that the residual part may not truly reflect the misspecification in 2MaxW or 2MaxB. Similar concerns have been raised for developing the method of MUML (Muthén, 1994) and for separately evaluating the within and between level structures (Yuan and Bentler, 2007). Since it is very unlikely to have a correct model specification in practice, results obtained for 2MaxB and 2MaxW may be too optimistic to generalize for empirical dataset. The performance of 2MaxB and 2MaxW models applied in substantial research warrants for future investigation. In addition, the model specification may become more complicated when there is more than one factor or when the observed variables are not normally distributed. Future study can be conducted to investigate the performance of 2MaxB and 2MaxW in more complex settings.

## Author contributions

JW designed the study, conducted the simulation study and took a leading role in writing the manuscript. JL, MN, and YH conducted a part of the simulation and prepared some tables and figures.

### Conflict of interest statement

The authors declare that the research was conducted in the absence of any commercial or financial relationships that could be construed as a potential conflict of interest.

## Footnotes

^1^The exemplary Mplus syntax of 2MaxB model is provided in **Appendix** for reader's reference.

^2^Because CPCSC is the maker variable so its factor loading would constantly fixed at one for all the analytical models. Therefore, we didn't present its bias measures. The bias measures for CPCSW had the same pattern with CPCSA; thus, we did not present the bias statistics, either.

^3^The 95% confidence interval coverage rate (95%, defined as the empirical proportion for which the 95% confidence interval of estimate contained the true population parameter value).

^4^Empirical power (Sig., defined as the empirical significance pattern of the estimates; that is empirical power = average rate of significant estimates over total replications).

^5^In two-level models, the variables are partitioned into level-l and level-2 components. so the *r*^2^ computed in these approaches should be interpreted as the proportion of variance in each within-group component that is accounted for by the lower-level model, and the proportion of variance in each between-group component that is accounted for by the higher-level model; while in 1-level mlr, *r*^2^ is proportion of variance in each indicators that is accounted for by an overall model where the variance composition is confounded by components from both levels.

## Appendix

### Mplus syntax for 2MaxB model

TITLE:    This is an example of a Maximum model
DATA:     FILE = Harter3_change_2l.dat;
VARIABLE: NAME = cpc31-cpc34 Cluster;
          USEVARIABLES = cpc31-cpc34;
          CLUSTER = Cluster;
ANALYSIS: TYPE = TWOLEVEL;
MODEL:
        %Within%                                ! Set up Within-level Model
        cpc3w BY cpc31@1 cpc32 cpc33            ! Specify lower-level CFA model
        cpc34;                                  ! Item residual variance
        cpc31 cpc32 cpc33 cpc34;                estimates
        %Between%                               ! Set up Between-level Model
        cpc31 WITH cpc32 cpc33 cpc34;           ! Estimate full rank
        cpc32 WITH cpc33 cpc34;                 ! variance-covariance matrix in
        cpc33 WITH cpc34;                       ! higher-level structure
        [cpc31 cpc32 cpc33 cpc34];              ! Item intercept estimates
OUTPUT: SAMP RES STAND MOD;
